# A Max-Flow Based Algorithm for Connected Target Coverage with Probabilistic Sensors

**DOI:** 10.3390/s17061208

**Published:** 2017-05-25

**Authors:** Anxing Shan, Xianghua Xu, Zongmao Cheng, Wensheng Wang

**Affiliations:** 1 School of Computer Science, Hangzhou Dianzi University, Hangzhou 310018, China; 141050025@hdu.edu.cn; 2 School of Science, Hangzhou Dianzi University, Hangzhou 310018, China; zmcheng@hdu.edu.cn; 3 College of Economics, Hangzhou Dianzi University, Hangzhou 310018, China; wswang@aliyun.com

**Keywords:** WSN, target coverage, connectivity, probabilistic sensor

## Abstract

Coverage is a fundamental issue in the research field of wireless sensor networks (WSNs). Connected target coverage discusses the sensor placement to guarantee the needs of both coverage and connectivity. Existing works largely leverage on the Boolean disk model, which is only a coarse approximation to the practical sensing model. In this paper, we focus on the connected target coverage issue based on the probabilistic sensing model, which can characterize the quality of coverage more accurately. In the probabilistic sensing model, sensors are only be able to detect a target with certain probability. We study the collaborative detection probability of target under multiple sensors. Armed with the analysis of collaborative detection probability, we further formulate the minimum *ϵ*-connected target coverage problem, aiming to minimize the number of sensors satisfying the requirements of both coverage and connectivity. We map it into a flow graph and present an approximation algorithm called the minimum vertices maximum flow algorithm (MVMFA) with provable time complex and approximation ratios. To evaluate our design, we analyze the performance of MVMFA theoretically and also conduct extensive simulation studies to demonstrate the effectiveness of our proposed algorithm.

## 1. Introduction

With wide deployment of wireless sensors in the real world, such as air quality monitoring and intrusion detection, wireless sensor networks (WSNs) have attracted tremendous research attention. Sensor coverage is one of the fundamental issues in WSNs. Sensors deployed under stochastic or manual deployment work cooperatively to accomplish a task such as sensing a certain field of interest. Based on the coverage subject, coverage issues can be classified into three categories: target coverage (e.g., [1,2,3,4,5,6]), area coverage (e.g., [7,8,9,10]) and barrier coverage (e.g., [11,12,13,14,15]). The objective of target coverage aims to activate a subset of sensor nodes to monitor targets, which randomly appear in the 2D plane. In the context of the detection application, a target usually represents a static object that periodically generates some event signals, such as an acoustic signal.

In general, WSNs contain one or more sinks, which collect data from sensor nodes. Two sensor nodes can communicate with each other directly if they are within their communication range. As further studies of target coverage, connected target coverage (CTC) includes a more practical constraint, i.e., all active sensor nodes in CTC must be connected to the sink (probably via some relaying nodes). In order to transmit sensing data to the sink, each sensor node must find a route to the sink. Under this restriction, the nodes can communicate with each other and exchange information constructing an ad hoc network. Associated with this issue, existing work focuses mainly on two categories of optimization problems.

One of them is to find the least cost of potential sensor nodes satisfying both coverage and connectivity from a given set of sensor nodes, such as [1,16,17,18]. We name this problem the minimum cost CTC problem. The CTC problem under both the Boolean sensing model and the probabilistic model has been proven to be NP-hard [1,18]. This strategy minimizes the overall network cost as covering targets with the minimum number of sensor nodes. Consequently, if expensive sensors remain as a constraining resource that is economically infeasible for substantial over provision, we should take the minimum number of sensors as an optimization objective.

Most studies are concerned with maximizing network lifetime (e.g., [19,20,21]) in the CTC problem as for sensors’ limited resources. The maximum lifetime CTC problem aims to schedule the activation of sensors to prolong network lifetime. To make a network last beyond the lifetime of an individual node, redundant nodes must be deployed. Hence, low-cost sensors with adequate energy may be the ideal basis for this issue.

In this paper, we study the CTC problem with omnidirectional probabilistic sensors working cooperatively. This is motivated by the observation that much work has been done to address the CTC problem; however, few of them take the probabilistic model (e.g., [1,16,17,21]) into consideration. Existing work makes a perfect assumption that a target will be covered when it lies in the sensing range of a sensor node (binary detection mode). This means that the event will be detected with probability 100%, either the target is located very close to the sensor, or it lies on the border of the sensing range. In practice, the sensing capability of a sensor is always affected by environmental factors, especially for acoustic sensors. Associated with the reasons mentioned above, the sensing quality of a sensor is represented by its detection probability, which follows a probabilistic distribution. Several empirical models have been proposed (e.g., [22,23]). Since the probabilistic sensing model utilizes a log-distance path loss model [23], it means that the signal attenuates over distance, and it can be detected with high probability closer to the sensor. Leveraging the sensing model from probabilistic sensors, we can characterize the quality of sensor coverage more accurately.

We begin by highlighting the challenges we face with existing mechanisms. Considering the CTC problem under the probabilistic sensing model, we define the minimum ϵ-connected target coverage (ϵ-CTC) problem. The objective of the problem is to activate the minimum static sensors to achieve the detection probability threshold ϵ for all of the targets. All sensors work in a cooperative fashion, which can improve the detection probability of all targets and reduce the number of active sensors impressively. Furthermore, all active sensors should retain connectivity with the sink (via the relaying node). The challenge of the minimum ϵ-CTC problem we face is how to activate as few sensors as possible, to achieve high detection threshold ϵ and provide connectivity for WSNs. Much different from sensors based on the 0/1 coverage model, probabilistic sensors have to cooperate with each other to achieve threshold ϵ. This means that two or more sensors must be activated for one target, while only one is needed in the 0/1 disk model. This results in the sharp growth of candidate activation schemes, while taking all targets into consideration simultaneously. As a result, it is a non-trivial task to determine a better scheme from a tremendous amount of candidate schemes. Besides, different activation schemes will bring in different costs in connectivity. This induces further complexities in finding candidate activation schemes, as we must take account of the cost for connectivity at the same time. Although the probabilistic model was adopted in [19,20], is has been proven inadequate for a variety of reasons. In [19], an unrealistic assumption is made that a target can be always detected with a probability over the detection threshold by at least one sensor, while the authors in [20] take mobile sensors into consideration. Essentially, both of them fail to schedule probabilistic sensors to detect targets in a cooperative fashion. In [18], an efficient algorithm probabilistic sensors coverage algorithm (PSCA) is presented based on set selection resulting in a better schedule scheme. However, PSCA is time consuming and unfeasible in large-scale WSNs.

To overcome the deficit of existing work where many redundant sensors are needed to achieve the unrealistic assumption in [19] and expensive mobile sensors are required in [20], we design an efficient algorithm with cost-effective static sensors to address the above challenges, inspired by the principle that sensors work in a collaborative way. The cost in [19,20] is high, since they fail to make sensors collaborate with each other. We firstly theoretically analyze the collaborative detection probability of a target by multiple sensors. Based on the theoretical analysis, we define the detection gain used to characterize a sensor’s influence on some targets. A target is deemed to achieve threshold ϵ when its cumulative detection gain by sensors exceeds a certain threshold. On the basis of graph theory, we map the minimum ϵ-CTC problem into a flow network. We show that the problem is NP-hard and propose a bounded approximation Algorithm 1, named the minimum vertices maximum flow algorithm (MVMFA). The key insight of MVMFA is that each augmenting path picked out by the pivotal Algorithm 2 FindPath has more flow and few inactive sensors. This means that a sensor with a high detection probability, but passing few relaying sensors by, will be activated firstly. Our main contributions are summarized as follows.

1. We formulate the minimum ϵ-CTC problem with omni-directional probabilistic sensors.
2. By reducing a minimum ϵ-detection coverage problem to the minimum ϵ-CTC problem, we prove it is NP-hard and transform it to the max-flow problem.
3. We propose the minimum vertices maximum flow algorithm to solve our problem and theoretically show its time complexity and approximation bound.

The rest of the paper is organized as follows. In Section 2, a review of the relevant literature is given. In Section 3, the problem statement and formulation are presented. Section 4 discusses the theoretical analysis of the problem. Section 5 presents our algorithm design and analysis. Simulation results are presented in Section 6, and finally, we conclude the paper in Section 7.

## 2. Related Work

WSNs have many applications in air quality, water pollution, intrusion detection, etc. [1,2,3,4,5,6,7,8,9,10,11,12,13,14,15,24]. Among the tremendous works in the research field of coverage, we survey the related CTC research proposals in the following section.

In earlier research related to CTC problem, the concept of connected coverage in target coverage in WSNs was proposed by Zhao in [25]. The objective of connected coverage is to maximize the network lifetime. They defined a maximum cover tree (MCT) problem, scheduling sensors into multiple sets. Each set represents a cover tree, which is rooted at the sink node that can cover all of the target points. The MCT problem is also NP-complete, and they provide an approximation algorithm. In [26], a round-based localized algorithm is proposed to coordinately determine sensor’ sensing range in order to prolong the WSN’s lifetime. However, in [26], only the sensors routed to the sink are required to be active, instead of all sensors in the backbone. Both target coverage and connectivity are satisfied in [25,26].

There are two primary approaches to classify existing research on the CTC problem. One is the type of optimization objective: the minimum cost CTC problem [1,16,17,27] or the maximum lifetime CTC problem [26,28,29,30]. Early work on the minimum cost CTC problem [1] assumed that the energy cost of WSNs included energy for both coverage and connectivity, called minimum-energy connected coverage (MeCoCo). By a reduction from the geometric set cover problem, the MeCoCo problem was proven to be NP-hard. The authors were the first to provide approximation algorithms with provable performance ratios for this problem. Similar to [1] except for the definition of the cost, the minimum cost CTC problem is addressed in [16,17], where the goals of the problem are to schedule the minimum sensors with the constraints of coverage and connectivity. Two approximate methods based on the local search technique and genetic algorithm, respectively, were provided in [16] with efficient results. In [17], an oppositional gravitational search algorithm (OGSA)-based approach was proposed to solve the similar problem in [16]. The simulation results show that the solution OGSA outperforms the approach of [16]. The objective of minimum cost CTC in [27,28] was to design an efficient algorithm to place minimum relaying nodes to provide desired k-connectivity. A genetic algorithm-based approach, as well as a greedy-based approach are proposed in [27] for minimum cost CTC, and a heuristic is designed for maximum lifetime in [28]. In [30], multiple sensing units are additionally taken into account. Two distributed heuristic schemes, REFS (remaining energy first scheme) and EEFS (energy efficiency first scheme), are proposed. Evaluations show that both of them can prolong the network lifetime effectively. However, EEFS outperforms REFS in network lifetime, but REFS is time saving.

Another way to classify existing work is by the type of sensing models. Some early works assumed that the sensing model was a 0/1 disk [16,28,29,30,31], while more recent work began to take probabilistic models into consideration [18,19,20,23,32,33,34]. An algorithm, CWGC-PM (communication weighted greedy cover probabilistic model), is specially designed to solve the CTC problem under the probabilistic coverage model in [19]. However, they make an unrealistic assumption that each target is always detected with a probability over the detection threshold by at least one of the active source sensors. In [20], mobile sensors are adopted to cover target beyond the detection threshold by moving closer to target. Probabilistic sensors were also employed to cover a series targets in [32,33]. A genetic algorithm based on a probabilistic coverage matrix is designed to select the minimum sensors to meet the probability of detection requirement in [32]. However, they fail to take account of connectivity. Additionally, probabilistic sensors were used in [33] to track moving targets. The probabilistic sensors coverage algorithm (PSCA) with provable approximation ratios [18] for the minimum ϵ-detection coverage problem aims to reduce the number of active sensors. They map the problem into a set select problem by constructing the candidate coverage set (CCS) and activate sensors for coverage requirement from the CCS. Moreover, a Steiner tree algorithm is used to picked out some sensors as relay nodes to maintain the network connectivity.

In summary, although probabilistic sensors have been adopted for the coverage problem [18,19,20,23,32,33,35], there are still some differences. The subject detected in [13,33,35] is different from us, since the work in [13] focuses on barrier coverage. The paper [33] is for moving targets tracking, and the paper [35] solves the area coverage problem [19]. Additionally, the authors in [23,32] fail to take connectivity into consideration. Despite that PSCA [18] could obtain a better performance in the number of active sensors, it is infeasible for large-scale WSNs because of the high time complexity. In this study, an approximate algorithm, MVMFA, is proposed to schedule the sensors, such that a given set of targets can be detected beyond the detection threshold ϵ, and the sensing information can be routed to the sink.

## 3. Preliminaries and Problem Formulation

In this section, we first describe the probabilistic sensing model in detail. After that, we present the network model for our research. This is followed by a formal statement of the minimum ϵ-connected target coverage problem. For convenience, the symbols in this paper are shown in Table 1.

### 3.1. Sensing Model

Targets are often modeled as a set of discrete space points within the sensor field. However in this paper, a target denotes a static object that generates some event signals periodically, such as the acoustic signal. Heterogeneous probabilistic sensors will be deployed to capture the occurrence of the event by receiving the signal from a target object. A target will obtain the coverage requirement when it can be detected by sensors beyond the detection probability threshold ϵ. Under this circumstance, an occurrence of the event will be captured beyond the minimum detection probability ϵ. In this study, the meaning of the target is the same as the target in [18].

**Figure 1:**
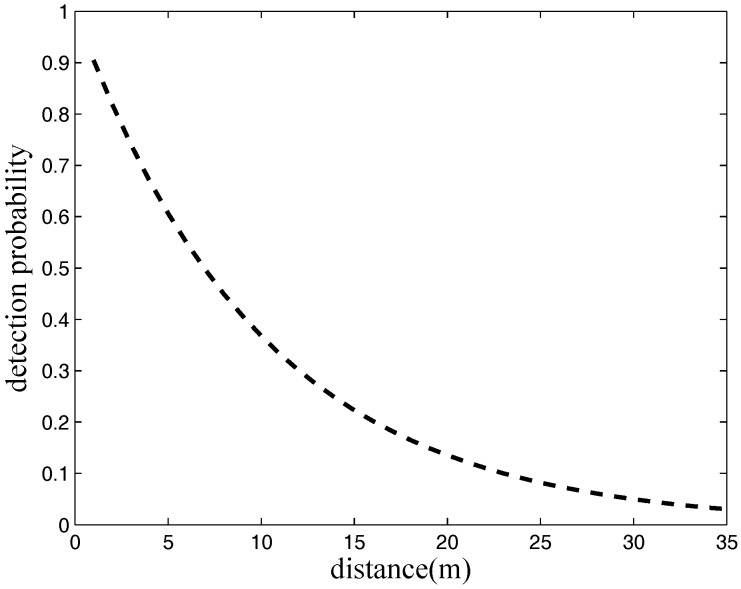
Change in detection probability with distance (m), where α=0.1.

In the probabilistic sensing model, sensors detect targets by received energy, which attenuates as the distance increases. As a result, probabilistic sensors, which can characterize the quality of coverage more accurately, are often used in the field of detection applications. The detection probability is a attenuation function p=λ(d), where *d* is the distance between sensors and targets. It is a continuously decreasing distance-dependent attenuation function. Several models have been proposed (e.g., [23,34,36]). For example, the work in [22] suggested that the detection probability of a location *t* by a sensor *i* can be characterized by:

(1) $$p_{i}\left(t\right)=\left\{\begin{array}{c} e^{-\alpha d(i,t)},\;if\;d\left(i,t\right)\leq r_{sk}\\ 0,\;otherwise \end{array}\right.$$

where α is a parameter representing the physical characteristics of the sensor, d(i,t) is the distance between *i* and *t* and $r_{sk}$ is the detection boundary of the probabilistic sensors. The detection probability is considered zero once the distance between some target and some sensor is beyond $r_{sk}$. Assuming that the energy radiated by targets is equal, Figure 1 describes the relationship between detection probability and distance, where α=0.1.

Multiple probabilistic sensors working in a cooperative fashion can enhance the detection probability impressively. Assuming that $s_{t}$ is the sensor set around the target *t*, the collaborative detection probability of *t* is denoted by P(t). P(t) is computed by the probability formula, which integrates the detection probability of each sensor in $s_{t}$, i.e.,

(2) $$P\left(t\right)=1-\prod_{i\in s_{t}}\left(1-p_{i}\left(t\right)\right)$$

**Figure 2:**
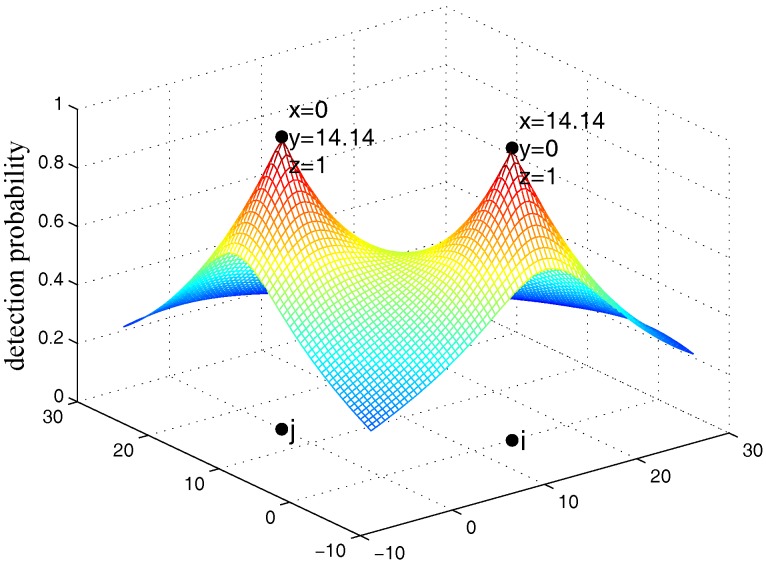
Change in collaborative detection probability with two sensors *i* and *j*.

Figure 2 presents the collaborative detection probability, for two sensors *i* and *j* located at (0,14.14,0) and (14.14,0,0), respectively. If an event occurs at the middle point between them, the collaborative detection probability is 0.60, while the individual detection probabilities for both *i* and *j* is 0.36.

Each sensor to be routed to the sink (probably via some relaying nodes) has a transmission radius $R_{t}$. Two sensors can communicate with each other within Euclidean distance $R_{t}$. We denote it as relaying sensor, which is only activated for communication.

### 3.2. Network Model

**Figure 3:**
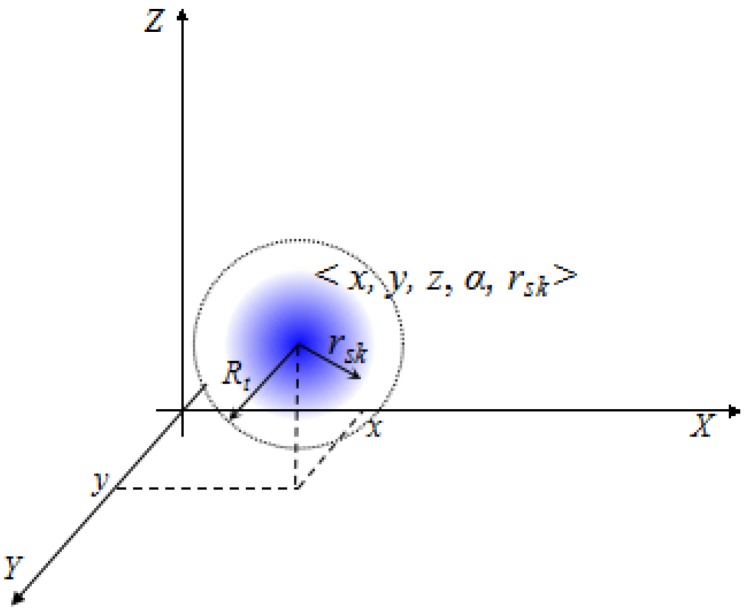
Omni-directional probabilistic sensor denoted by a four-tuple.

Our WSNs with static probabilistic sensors will be deployed in an *L* × *L* 2D Euclidean plane under stochastic deployment. As Figure 3 shows, a probabilistic sensor is denoted by four-tuple $<x,y,\alpha ,r_{sk}>$, where x,y is the coordinate in the 2D plane; α and $r_{sk}$ are intrinsic parameters of the sensor as the previous section mentioned. All sensors are heterogeneous in terms of α and $r_{sk}$, but share the same transmission radius $R_{t}$. Let *S* denote the sensor set, and *D* denote the target set. All targets appear randomly in the same plane: D∩S = ø. Each target can be detected by multiple sensors nearby, and vice versa. We assume that the location information of sensors and targets can be obtained by some localization methods previously. There also exists a sink node, and all active sensors must be routed to it.

### 3.3. Problem Statement

The CTC problem requires that the detection probability of each target is at least ϵ by activating sensors from the randomly-deployed *S*. Given the detection probability threshold ϵ, we formally define the minimum ϵ-connected target coverage problem based on our sensing model and network model as follows.

Minimum ϵ-connected target coverage problem (minimum ϵ-CTC problem): Given a set of sensors *S*, a set of targets *D* and a sink S, targets in *D* randomly distributed are required to be detected with the minimum detection probability ϵ. We aim to activate a subset C⊆S with the least sensors that the detection probability of each target in *D* detected by sensors in *C* is at least ϵ. In addition, all sensors in *C* need to be routed to the sink S (via relaying sensor nodes).

Indeed, it is a challenge to find the optimum solution for the minimum ϵ-CTC problem in polynomial time.

## 4. Theoretical Analysis

For mapping our problem into a network flow problem, we first linearize the collaborative detection probability formula similar to [13,18]. Next, we prove that the minimum ϵ-CTC problem is NP-hard by a reduction from the minimum ϵ-detection coverage problem. Lastly, we describe the transformation from our problem to a network flow problem in detail and present a method to set $r_{sk}$.

### 4.1. Analysis of Detection Probability

Given a target *t* detected by a sensor set $s_{t}$, the detection probability is Equation (2). According the coverage requirement, P(t) should be larger than ϵ. Then, we can get:

$$P\left(t\right)=1-\prod_{i\in s_{t}}\left(1-p_{i}\left(t\right)\right)\geq \epsilon$$

We linearize the formula as follows.

$$P\left(t\right)=1-\prod_{i\in s_{t}}\left(1-p_{i}\left(t\right)\right)\geq \epsilon$$

$$\Rightarrow 1-\epsilon \geq \prod_{i\in s_{t}}\left(1-p_{i}\left(t\right)\right)$$

$$\Rightarrow \ln\left(1-\epsilon \right)\geq \sum_{i\in s_{t}}\ln\left(1-p_{i}\left(t\right)\right)$$

$$\Rightarrow -\ln\left(1-\epsilon \right)\leq -\sum_{i\in s_{t}}\ln\left(1-p_{i}\left(t\right)\right)$$

The term Ψ=−ln(1−ϵ) is defined as the aggregate gain threshold.

Sensor detection gain $\phi_{i}\left(t\right)$: A target can get detection gain from sensor *i* according to the formula:

(3) $$\phi_{i}\left(t\right)=-\ln\left(1-p_{i}\left(t\right)\right)$$

The detection gain reflects how much impact a sensor has on one target.

Cumulative detection gain $\sum_{i\in s_{t}}\phi_{i}\left(t\right)$: A target’s cumulative detection gain is to aggregate detection gains from the surrounding sensors.

Obviously, if the target *t* satisfies the coverage requirement, then its cumulative detection gain must be larger than Ψ, i.e.,

(4) $$\sum_{i\in s_{t}}\phi_{i}\left(t\right)\geq \Psi$$

### 4.2. NP-Hardness

In this section, we prove the NP-hardness of the minimum ϵ-CTC problem theoretically by a reduction from the minimum ϵ-detection coverage problem [18]. We have previously proven that the minimum ϵ-detection coverage problem is NP-hard without taking connectivity into consideration. According to the proven NP-hardness, for any instance in the minimum ϵ-detection coverage problem, it should be reduced to the minimum ϵ-CTC problem in polynomial time.

**Theorem** **1.** The minimum ϵ-CTC problem is NP-hard.

**Proof** **of** **Theorem** **1.** Assume an instance of the minimum ϵ-detection coverage problem, $S^{\prime }=\left\{1^{\prime },2^{\prime },...,n^{\prime }\right\}$ and $D^{\prime }=\left\{t_{1}^{\prime },t_{2}^{\prime },...,t_{m}^{\prime }\right\}$, where $S^{\prime }$ denotes the sensor set and $D^{\prime }$ denotes the target set. It aims to activate the least sensors from $S^{\prime }$ that all targets in $D^{\prime }$ must be detected beyond the detection probability threshold ϵ. Note that all sensors in the minimum ϵ-detection coverage problem share the same parameters. The reducing procedure will be presented in detail.

**Figure 4:**
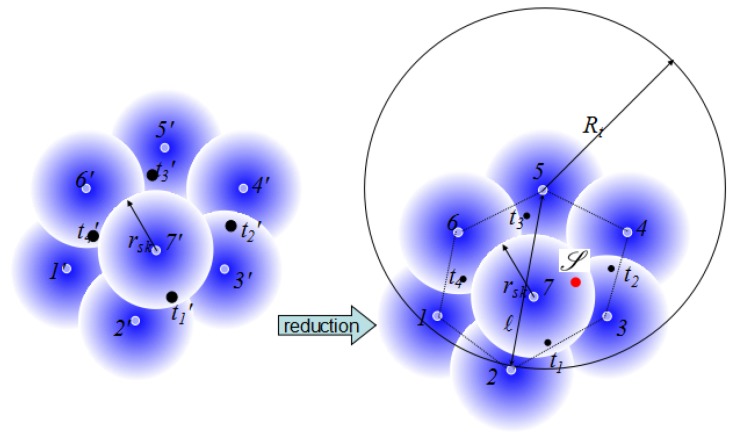
A reduction from the minimum ϵ-detection coverage problem to the minimum ϵ-CTC problem. Indeed, each sensor after the reduction has a communication radius. For simplicity, we draw only one sensor with the communication radius.

As shown in Figure 4, we calculate the diameter (denoted by *ℓ*) of the sensor set $S^{\prime }$ based on the rotating calipers [37] in O(n) time, followed by computing the convex hull of $S^{\prime }$. For each sensor $i^{\prime }$ in $S^{\prime }$, a corresponding probabilistic sensor *i* is created at the same location with the same $r_{sk}$ and $R_{t}=\ell$. Additionally, for each target $t_{i}^{\prime }$ in $D^{\prime }$, a corresponding target $t_{i}$ is put at the same location. Lastly, we put the sink S anywhere in the convex hull. As a result, any active sensor can communication with the sink directly with one hop. Under these settings, the minimum ϵ-CTC problem is to find the least probabilistic sensors with $r_{sk}$ and $R_{t}$, centered in sensors $\left\{1^{\prime },2^{\prime },...,n^{\prime }\right\}$ to detect all targets, which is exactly the same as the minimum ϵ-detection coverage problem.

In other words, the minimum ϵ-detection coverage problem is a special case of the minimum ϵ-CTC problem, under the constraints of homogeneous sensors with the same $r_{sk}$ and $R_{t}=\infty$. In [18], the minimum ϵ-detection coverage problem has been proven NP-hard. Therefore, the minimum ϵ-CTC problem must also be NP-hard. ☐

### 4.3. Problem Transformation

Both the detection probability and detection gain will be very small when the distance between sensor and target is large. Furthermore, it will be more cost-effective to obtain the probability with short distance.

Minimum detection probability $p_{min}$: $p_{min}$ is a pre-defined threshold set by applications. If the detection probability of a target *t* is detected by one sensor *i* to be less than $p_{min}$, we take it as zero, otherwise $e^{-\alpha d(i,t)}$.

We have previously presented a method in [18] to determine the $p_{min}$ for different detection probability threshold ϵ. Here, we give a suggestion to design the $r_{sk}$ for each probabilistic according to:

$$e^{-\alpha r_{sk}}=p_{min}$$

$$r_{sk}=\frac{-\ln p_{min}}{\alpha }$$

Due to requirements of both coverage and connectivity, the minimum ϵ-CTC problem is complex. We introduce a network flow model to solve it.

Based on the analysis of detection probability, we further build a flow graph G=(S∪D∪{S}∪{S},E) to characterize the feature of the network where *S* denotes the sensor set, where *D* is the target set; S denotes the sink; S represents the super source we created; and *E* is the edge set. In order to transform our problem to a max-flow problem, we create a super source S as the source of the flow graph *G*.

The construction of *G* is as follows:

(1) For ∀t∈D, we add directed edge 〈S,t〉 into *E* with capacity Ψ. We name it the virtual edge.
(2) For ∀t∈D, ∀i∈S, if $p_{i}\left(t\right)>p_{min}$, it will be linked with an directed edge 〈t,i〉 with capacity $\phi_{i}\left(t\right)$ according to Equation (3). We denote it the sensing edge.
(3) For $\forall i^{\prime }\in S,\forall i^{\prime \prime }\in S$, if $d\left(i^{\prime },i^{\prime \prime }\right)<R_{t}$, an undirected edge $\left(i^{\prime },i^{\prime \prime }\right)$ will be added into *E* and its capacity is +∞. We denote it the communication edge.
(4) For ∀i∈S, if $d\left(i,S\right)<R_{t}$, then 〈i,S〉∈E with capacity +∞.

**Figure 5:**
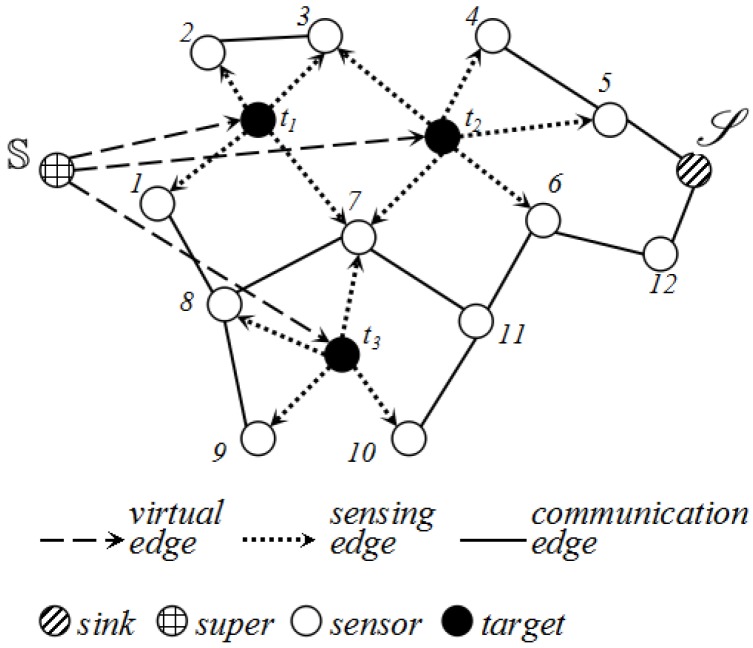
An example of the flow graph.

We show an example of *G* in Figure 5.

**Theorem** **2.** *When G’s maximum flow equals |D|×Ψ, the sensors delivering the maximum flow satisfy both the coverage and connectivity requirements for the minimum ϵ-CTC problem.*

**Proof** **of** **Theorem** **2.** When the maximum flow reaches |D|×Ψ, it means that each virtual edge is saturated. As a result, the flow from each target is Ψ, and it will be transferred to the sink S by communication edges. Assuming for each, $t\in D,s_{t}$ denotes the sensor set that transfers flow from *t*, we can get $\sum_{i\in s_{t}}\phi_{i}\left(t\right)\geq \sum_{i\in s_{t}}f(i)=\Psi$ (f(i) is the flow value through node *i*). As a result, the detection probability of *t* is at least ϵ based on Equation (4). Since Ψ flows from *t* will eventually arrive at the sink S, each sensor in $s_{t}$ can communicate with the sink (probably via some relaying nodes). ☐

According to Theorem 2, we can reduce the minimum ϵ-CTC problem to the minimum vertices maximum flow problem.

Minimum vertices maximum flow problem: Based on the flow graph G=(S∪D∪{S}∪{S},E) we constructed, our ultimate aim is to find a minimum set C⊆S, and the subgraph of the max flow of $G^{\prime }=\left(C\cup D\cup \left\{S\right\}\cup \left\{S\right\},E^{\prime }\right)$ (the $G^{\prime }$ construction method is the same as *G*) is equal to |D|×Ψ.

Without a doubt, the minimum vertices maximum flow problem is also NP-hard.

## 5. Algorithm Design

Based on the above analysis, we have proven the minimum vertices maximum flow problem to be NP-hard. It is hard to find the optimal solution in polynomial time. In this section, we first design an approximation solution, named the minimum vertices maximum flow algorithm (MVMFA). Next, the time complexity and approximation ratios are proven in theory.

### 5.1. Approximation Algorithm

The minimum vertices maximum flow problem aims to find a minimum set C⊆S to meet upper flow value |D|×Ψ. We design the MVMFA (Algorithm 1) based on the FindPath, which aims to find the augmenting path.

Firstly, we create flow graph *G* as mentioned in Section 4. As we know, the key to solve the problem using the network flow is to map the problem into the network flow graph. MVMFA is based on the classical network flow method of Ford–Fulkerson. The basic idea of MVMFA is to find the augmenting path iteratively with a bigger ρ:

$$\rho =\frac{augmenting\;path\;flow}{the\;number\;of\;inactive\;sensor\;nodes}$$

and the flow will be sent to the sink S along the path. The distinction between MVMFA and other maximum flow algorithms, such as Edmonds-Karp [38] and Dinic [39], is the method to find the augmenting path. Probabilistic sensors will be activated along the augmenting path.

**Algorithm 1** MVMFA.1:Createasupersources
2:CreateG=(S∪D∪{S}∪{S},E)
3:C=∅
4:callFindPathtofindapathμ(S,S)withabiggerρ
5:**if** flowofμ(S,S)is0 **then**
6:    **return *C***
7:**else**
8:    sendtheflowalongtheaugmentingpathμ(S,S)
9:    activatethesensornodesinμ(S,S)andputthemintoC
10:    **goto 4**
11:**end if**


### 5.2. Algorithm Analysis

We analyze the performance of our proposed algorithm MVMFA theoretically in this section.

In this paper, we present the FindPath algorithm (Algorithm 2) to find an augmenting path μ(S,S). FindPath is based on the priority queue and breadth-first-search method. We define the structure NodeInfo to record the information of the search nodes.

**struct** NodeInfo{

 flow;

 count;

 id;

 NodeInfo*father;

};

As mentioned above, flow represents the flow value, while count is the number of inactive sensor nodes when achieving some search node. Id is the sensor identifier corresponding to the search node. In the priority queue we defined, the search node with bigger ρ will be in front of the queue (if the count is zero, the ρ with bigger flow is larger than the others).

**Algorithm 2** FindPath.1:createapriority_queue〈NodeInfo*〉Q;
2:createaninitialsearchnodewhereflow=0,id=S,count=0andfather=NULL;
3:pushtheinitialsearchnodeintoQ;
4:bFindPath=FALSE;
5:**while** Qisnotempty **do**
6:    pop the first search node pnode of Q;7:    **if** pnode→idisS **then**
8:        bFindPath=TRUE;
9:        calculatetherouteμ(S,S)bythefather;
10:        returnμ(S,S);
11:    **end if**
12:    u=pnode→id;
13:    **if** uisunvisited **then**
14:        setuvisited;
15:    **else**
16:        continue;
17:    **end if**
18:    **for** eachadjacentvertexiofu **do**
19:        **if** iisnotvisitedandc〈u,i〉>0 **then**
20:           $create\;a\;new\;search\;node{\;}^{\prime }new^{\prime }$
21:           **if** iisactive **then**
22:               new→count=pnode→count;23:           **else**
24:               new→count=pnode→count+1;25:           **end if**
26:           new→id=i;27:           new→flow=min(c〈u,i〉,pnode→flow);28:           new→father=pnode;29:           Q.push(new);30:        **end if**
31:    **end for**
32:**end while**
33:**if** bFindPathisFALSE **then**
34:    return0;
35:**end if**

**Lemma** **1.** *After each invocation of FindPath, some sensing edge or virtual edge will be saturated.*

**Figure 6:**
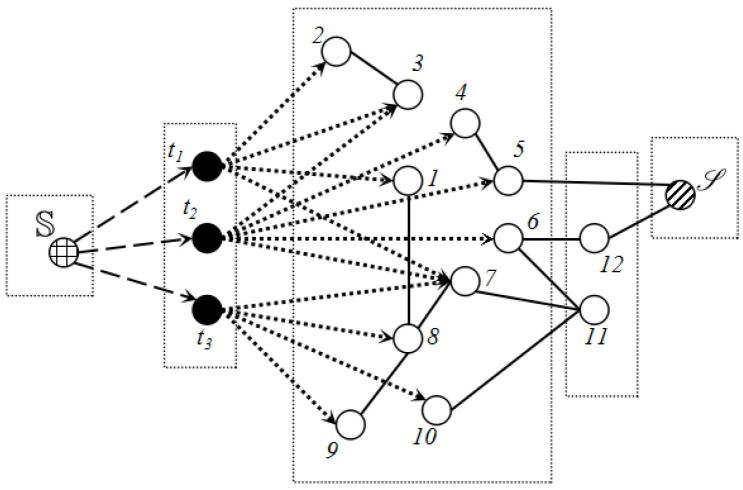
A flow graph can be divided into five parts: super source, target nodes, sensing nodes, relaying nodes, sink node.

**Proof** **of** **Lemma** **1.** All vertices in *G* can be divided into five parts as Figure 6 shows, super source, target nodes, sensor nodes, relaying nodes and the sink S. The characteristic of our flow graph *G* determines that the flow in μ(S,S) starts from the virtual edge, passes the sensing edge and arrives at the sink through the communication edge. The flow value of each augmenting path relies on the capacity of the virtual edge and sensing edge due to the limited capacity.

Before we first call FindPath, all sensor nodes have not been activated. Assuming μ(S,S)=〈S,t,a,b,c,...,S〉 is the first augmenting path by FindPath, 〈S,t〉 denotes virtual edge, while 〈t,a〉 represents the sensing edge. It is obvious that the flow in μ(S,S) is $min(c\left(S,t\right),\phi_{a}\left(t\right))$ (c(S,a) is the capacity of 〈S,a〉). Thus, 〈S,a〉 or 〈t,a〉 will be saturated. In either case, 〈t,a〉 will not appear in the augmenting path any more.

After that, the augmenting path $\mu^{\prime }\left(S,S\right)=\langle S,t^{\prime },a^{\prime },b^{\prime },c^{\prime },...,S\rangle$ that FindPath explores later has two scenarios:

(1) $\mu^{\prime }\left(S,S\right)$ does not contain activating sensor nodes. This scenario is the same as μ(S,S), and $\langle t,a^{\prime }\rangle$ will not be selected by FindPath anymore.

(2) $\mu^{\prime }\left(S,S\right)$ contains activating sensors. Without loss of generality, we assume $c^{\prime }$ is the first active sensor node, which is in $\mu^{\prime }\left(S,S\right)$. There is no doubt that flow in $\mu^{\prime }\left(S,S\right)$ equals $min(c\left(S,t^{\prime }\right),\phi_{a^{\prime }}\left(t^{\prime }\right))$. Due to the active sensor node $c^{\prime }$, there must be a path $\mu (c^{\prime },S)$ in which all sensor nodes have to be activated previously. Along $\mu (c^{\prime },S)$, flow will not decrease while the count of the search node will not increase. Whether the flow is $c(S,t^{\prime })$ or $\phi_{a^{\prime }}\left(t^{\prime }\right)$, FindPath will not pass $\langle t^{\prime },a^{\prime }\rangle$ anymore. In summary, after each invocation of FindPath, some sensing edges or virtual edges must be saturated. Meanwhile, FindPath passes every sensing edge at most once. ☐

**Theorem** **3.** *The time complexity of MVMFA is $O(\kappa |D|\times |E{|}^{2}\times \log\left|E\right|)$, where κ is the maximum out-degree of targets.*

**Proof** **of** **Theorem** **3.** Lemma 1 shows that each sensing edge is selected at most once. Hence, FindPath is invoked at most $\sum_{t=1}^{\left|D\right|}out-degree\left(t\right)$ times (out−degree(t) denotes the out-degree of target *t*). In FindPath, the while loop executes at most O(|E|) times because each edge is pushed into the priority queue at most once (each search node represents an edge). In the while loop, it costs O(|S|log(|E|)) time to push adjacent search nodes into the priority queue. Therefore, the time complexity of MVMFA is $O(|S|log(|E|))\times O(|E|)\times \sum_{t=1}^{\left|D\right|}out-degree\left(t\right)=O(\kappa |D|\times \left|E|\log(|E|)|S|\right)$. ☐

We assume *N* is the size of *C* computed by MVMFA, while $N_{opt}$ is the optimum.

**Theorem** **4.** *$\frac{N}{N_{opt}}\leq \beta h_{max}\frac{\ln(1-\epsilon )}{ln(1-p_{min})}$, where $h_{max}=\max_{t\in D}hop\left(t,S\right)$ (hop(a,b) is the minimum hops from a to b minus one) and β is the maximum indegree of sensor nodes.*

**Proof** **of** **Theorem** **4.** Assuming MVMFA invokes *k* times FindPath, each flow value is denoted by $\phi_{1},\phi_{2},...\phi_{k}$, and each count is $\Delta_{1},\Delta_{2},...\Delta_{k}$, respectively.

According to FindPath based on the priority queue and the breadth-first-search, we can find the augmenting path with a bigger ρ. We can get:

(5) $$\begin{array}{c} \frac{\phi_{1}}{\Delta_{1}}\geq \frac{\phi_{min1}}{{\Delta_{1}}^{\prime }}\\ \frac{\phi_{2}}{\Delta_{2}}\geq \frac{\phi_{min2}}{{\Delta_{2}}^{\prime }}\\ ...\\ \frac{\phi_{k}}{\Delta_{k}}\geq \frac{\phi_{mink}}{{\Delta_{k}}^{\prime }} \end{array}$$

where in the *i*-th invocation of the FindPath, $\phi_{mini}$ denotes the minimum flow of all search nodes and ${\Delta_{i}}^{\prime }$ represents the maximum count. In the worst case scenario, the augmenting path may arrive at the sink with the flow value $\phi_{mini}$ and ${\Delta_{i}}^{\prime }$ inactive sensors. Furthermore, ${\Delta_{i}}^{\prime }$ is not larger than $h_{max}$, i.e.,

(6) $${\Delta_{i}}^{\prime }\leq h_{max}$$

with Equations (5) and (6),

(7) $$\frac{\phi_{i}}{\Delta_{i}}\geq \frac{\phi_{mini}}{h_{max}}\geq \frac{-\ln(1-p_{min})}{h_{max}},i=1,2,...k$$

According to Equations (5) and (7), we can get:

$$\frac{\phi_{1}+\phi_{2}+,...,+\phi_{k}}{\Delta_{1}+\Delta_{2}+,...,+\Delta_{k}}\geq \frac{-\ln(1-p_{min})}{h_{max}}$$

$$\frac{|D|\times \Psi }{N}\geq \frac{-\ln(1-p_{min})}{h_{max}}$$

(8) $$N\leq \frac{\left|D|\ln(1-\epsilon \right)}{\ln(1-p_{min})}\times h_{max}$$

The minimum ϵ-CTC problem concentrates on finding the minimum *C*. When we just take account of coverage without connectivity, we assume $N_{c}$ is the minimum number sensors ensuring coverage needs. Obviously, $N_{opt}\geq N_{c}$. Let β denote the maximum indegree of sensor nodes. It means one sensor can detect at most β targets. Therefore, the least number of sensors we need satisfies:

(9) $$N_{opt}\geq N_{c}\geq \frac{\left|D\right|}{\beta }$$

With Equations (8) and (9),

$$\frac{N}{N_{opt}}\leq \frac{\ln\left(1-\epsilon \right)|D|h_{max}}{\ln\left(1-p_{min}\right)\times \frac{\left|D\right|}{\beta }}=\beta h_{max}\frac{\ln(1-\epsilon )}{\ln(1-p_{min})}$$

☐

## 6. Performance Evaluation

In order to illustrate the effectiveness and efficiency of our new method, we present the simulation results. At the same time, we compare our algorithm with the minimum weight barrier algorithm (MWBA) [13], the localized coverage quality algorithm (LoCQAL) [20] and PSCA [18]. In our simulation studies, static probabilistic sensors are randomly deployed in different size areas, with the constraint that each target is detected at least ϵ. We predefine the $p_{min}$ equal to 0.2 for all simulations. Furthermore, we randomly assign the parameter α for each sensor, ranging from 0.8 to 1. However, we adjust the parameter $r_{sk}$ for each sensor with the constraint of $p_{min}=0.2$. This paper adopts the exponential attenuation probabilistic model proposed in [22].

### 6.1. Algorithm Evaluation

**Figure 7:**
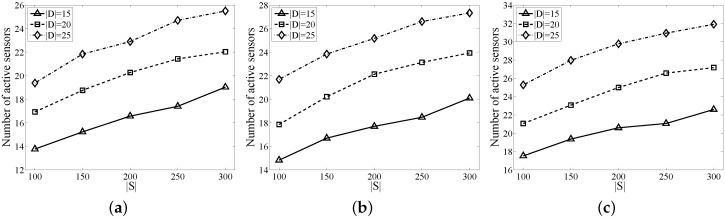
Performance of the minimum vertices maximum flow algorithm (MVMFA) with different |S| and |D|. (**a**) ϵ=0.7; (**b**) ϵ=0.8; (**c**) ϵ=0.9.

As shown in Figure 7, extensive simulations are conducted to evaluate the performance of MVMFA, in terms of detection region size *L*, number of targets |D| and detection threshold ϵ. The number of sensors |S| ranges from 100 to 300. For a given |S|, we randomize the sensors in a square of L×L=75|S|m^2^ plane and generate different target sizes to calculate the corresponding active sensors. All sensors have a communication radius $R_{t}=40$ m. We repeat each experiment 30 times in the same scenario and compute the average as the result for different parameters.

The purpose of the minimum ϵ-CTC problem is to minimize the number of activated probabilistic sensors, including the relaying sensors. Figure 7a–c show the number of sensors activated by MVMFA under different detection thresholds ϵ, 0.7, 0.8 and 0.9. Different lines correspond to different target sizes |D|(15, 20 and 25). Three remarkable observations are: (i) the algorithm has a better stability with the target size |D| increasing; (ii) the bigger the detection probability threshold ϵ, the larger the number of the active sensors is; and (iii) given the requirements of both coverage and connectivity, increasing the network size brings the number of active sensors up.

**Figure 8:**
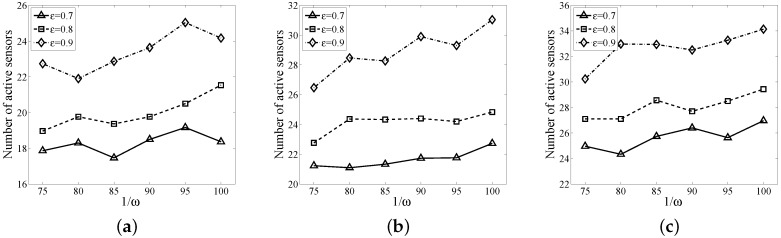
Performance of MVMFA with different sensor densities ω and ϵ. (**a**) |D|=15; (**b**) |D|=20; (**c**) |D|=25.

In Figure 8, we evaluate the performance of MVMFA with respect to the density $\omega =\frac{\left|S\right|}{L\times L}$ and ϵ. We fix the length of the sensor field L=150 m and generate ω×L×L sensors, the number of targets being 15, 20 and 25, respectively. We vary $\frac{1}{\omega }$ to 75, 80, 85, 90, 95, 100, respectively, and vary ϵ to 0.7, 0.8, 0.9, respectively. Figure 8 shows the number of active sensors with different densities and ϵ. As expected, the number of active sensors increases with larger $\frac{1}{\omega }$ and ϵ, respectively.

### 6.2. Comparison of the Algorithms

In this subsection, we first compare the performance of MVMFA with that of MWBA (minimum weight barrier algorithm) [13]. As we mentioned in the Related Work section, MWBA is designed to solve barrier coverage with the probabilistic sensors, aiming to prolong the lifetime of WSNs. One of the key differences is the method used to construct the network flow graph. However, under some modifications of the graph construction in MWBA, it can be used to solve our problem. To apply MWBA to our problem, we adopt FlowOrientedMWBA with the constraint of each sensor’s weight being one.

**Figure 9:**
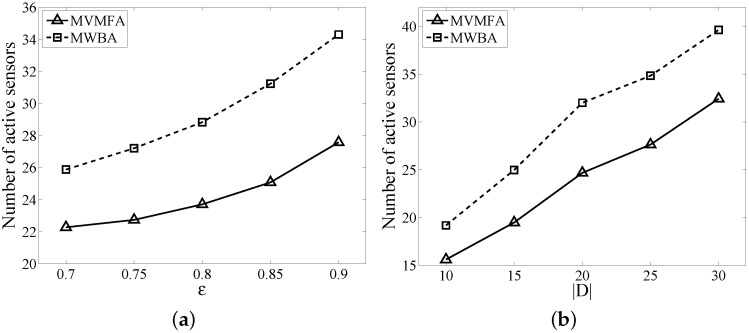
Impacts of ϵ (**a**) and |D| (**b**) to MVMFA and the minimum weight barrier algorithm (MWBA).

The number of sensors achieved by MVMFA and MWBA versus different ϵ and |D| is plotted in Figure 9. We fix the network size |S| to 300 and $R_{t}$ to 40 m, while varying the detection probability ϵ in Figure 9a and the target size in Figure 9b. The target size is set to 20 in Figure 9a, and the detection probability threshold is 0.8 in Figure 9b. The plots suggest that MVMFA always has a better performance compared with MWBA in different scenarios. MVMFA uses FindPath to find the augmenting path, while MWBA invokes Breadth First Search (BFS) in the Edmonds–Karp algorithm. The FindPath always finds a better augmenting path with bigger ρ (more flows and less inactive sensors), whereas BFS fails to take the state of sensors (inactive or active) into consideration. It hence naturally outperforms MWBA.

**Figure 10:**
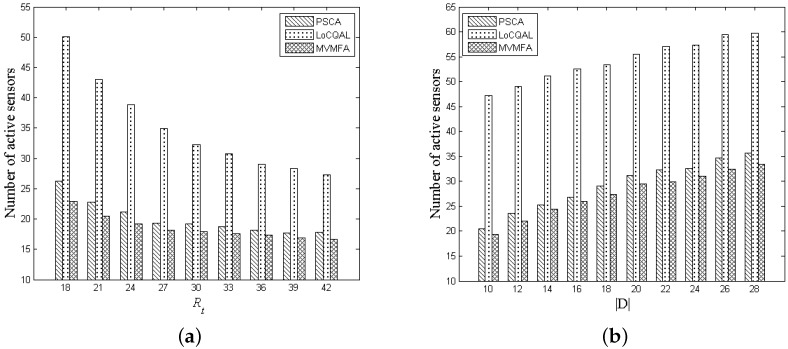
Performance of PSCA, the localized coverage quality algorithm (LoCQAL) and MVMFA with different $R_{t}$ (**a**) and |D| (**b**).

As shown in Figure 10, the methods considered include our new method MVMFA, the probabilistic sensor coverage algorithm (PSCA) in [18], the localized coverage quality algorithm (LoCQAL) in [20]. In order to apply the LoCQAL method to our problem, the probabilistic sensors in the LoCQAL method is set static. We randomly deploy 150 sensors with the detection probability threshold ϵ 0.8 in this comparison experiment.

We vary the communication radius $R_{t}$ ranging from 18 m to 42 m in Figure 10a and also simulate different application scenarios by varying target size |D| ranging from 10 to 28 in Figure 10b. With a larger communication radius $R_{t}$, we observe less sensors being activated in WSNs. This is because a larger communication radius $R_{t}$ leads to fewer relaying sensors being activated in connectivity. As shown in Figure 10b, more sensors will be activated with the target size increasing. In addition, it is obvious that our method is more efficient than both the PSCA method and the LoCQAL method. This is because in LoCQAL, the relaying nodes in the connected domination set (CDS) are always activated. Nevertheless, only the sensors routed to the sink are required to be active in our method MVMFA and PSCA, instead of all sensors in the CDS. The PSCA method is similar to our approach MVMFA in terms of performance, but it has a high time complexity. Since an enumeration algorithm called the candidate coverage set algorithm was used in PCSA, it is infeasible in large-scale WSNs.

## 7. Conclusions

In this paper, we study the minimum ϵ connected target coverage problem in WSNs. We aim to capture occurrences of the events by receiving the signal from a target object. We adopt omni-directional probabilistic sensors with the exponential attenuation probabilistic model. Based on the theoretical analysis of the probabilistic model, we propose the minimum ϵ-CTC problem with the NP-hard proof. In order to solve the problem, we map the minimum ϵ-CTC problem into a maximum flow problem with an extra optimization objective. We prove the MVMFA approach with provable time complexity and approximation ratios. Extensive simulation studies are conducted to evaluate our method, and the results demonstrate the effectiveness of our proposed algorithm.

In this work, we have neglected the energy consumption of different sensors and focus on the least number of activating sensors. However, there also exist sensors that are activated as relaying nodes. As just for connectivity, the relaying nodes consume less energy. In our future work, we will focus on minimizing the total energy cost of both coverage and connectivity. On the other hand, sensors with directional sensing ability are increasingly adopted for energy conservation. However, existing proposals mostly assume omni-directional probabilistic sensors. This motivates us to investigate the connected target coverage problem under directional probabilistic sensors. The evaluation of our algorithm in the real test-bed is also a meaningful work in the future. 

## Figures and Tables

**Table 1:** Summary of notations.

Symbol	Description
*S*	the sensor set
*D*	the target set
$p_{i}\left(t\right)$	the detection probability of target *t* by sensor *i*
P(t)	the collaborative detection probability of target *t*
$\phi_{i}\left(t\right)$	the detection gain of target *t* from sensor *i*
ϵ	the detection probability threshold
Ψ	aggregate gain threshold
$p_{min}$	the minimum detection probability threshold
S	the sink node
S	the super source of the flow graph *G*
